# Study protocol for a process evaluation of a cluster randomised controlled trial to reduce potentially inappropriate prescribing and polypharmacy in patients with multimorbidity in Irish primary care (SPPiRE)

**DOI:** 10.12688/hrbopenres.12920.2

**Published:** 2020-01-21

**Authors:** Karen Kyne, Caroline McCarthy, Bridget Kiely, Susan M. Smith, Barbara Clyne, SPPiRE Study team

**Affiliations:** 1 Department of General Practice, Royal College of Surgeons in Ireland, Dublin, Ireland

**Keywords:** Multimorbidity, Polypharmacy, Deprescribing, Randomised Controlled Trial, Process evaluation

## Abstract

**Background: **Multimorbidity (the presence of two or more chronic conditions) is associated with poorer health outcomes, particularly for patients with significant polypharmacy (≥15 medications), due to the higher risk of adverse events and drug interactions. The SPPiRE study will assess the effectiveness of a complex intervention to support general practitioners (GPs) to reduce potentially inappropriate prescribing and consider deprescribing in older people with multimorbidity and significant polypharmacy. The aim of the SPPiRE process evaluation is to understand how and why the intervention is effective or ineffective and to explore the potential for system wide implementation of the intervention using the Medical Research Council general themes of context, implementation and mechanism of impact.

**Methods: **The SPPiRE study is a clustered randomised controlled trial (RCT), aiming to recruit 55 general practices and 400 patients (≥65 years) on ≥15 medications throughout the Republic of Ireland.

This mixed-methods process evaluation of the SPPiRE study will integrate both quantitative and qualitative data. Quantitative data will be collected on use of the intervention elements and from GP questionnaires. Qualitative data will be collected from semi-structured telephone interviews with all intervention GPs and a purposeful sample of patients from intervention practices. The topic guide will explore barriers and facilitators to participation and implementation of the intervention.

Quantitative data will be analysed using descriptive statistics. Interviews will be transcribed and analysed using thematic analysis. Quantitative and qualitative data will be then be integrated.

**Discussion:** The SPPiRE cluster RCT will provide evidence regarding the effectiveness and practicability of delivering a structured medication review in reducing polypharmacy and potentially inappropriate prescribing for patients with multimorbidity. This process evaluation will provide information on how the intervention was implemented, how it was or was not effective and the potential for a system wide implementation.

**Trial registration:** ISRCTN 12752680, registration: 20/10/2016

## Background

Complex interventions involve a number of interacting components and often offer a degree of flexibility, or tailoring to the local environment ^1^. Therefore, it is often difficult to ascertain why a complex intervention was or was not effective, and if effective, what components of the intervention were responsible for that effect. The Medical Research Council (MRC) advise performing a process evaluation alongside the effectiveness evaluation of a complex intervention to assess how it was implemented, how it caused change and how the intervention interacted with the context in which it was implemented ^2^. This provides important information for policy makers on how a complex intervention might be implemented more widely into the healthcare system. A framework to guide process evaluations designed for cluster randomised controlled trials ^3^ advises that process evaluations should clearly state their purpose and research questions and as recommended by the MRC guideline, this paper sets out to pre-specify our process evaluation research questions and methods.

### Supporting prescribing in multimorbidity in primary care (SPPiRE)

There is a growing consensus that the current single disease framework is not appropriate when managing patients with multiple chronic conditions or multimorbidity, and that adhering to multiple single disease guidelines may lead to significant polypharmacy and inappropriate treatment burden for patients ^4^. The National Institute for Clinical Excellence (NICE) multimorbidity guideline advises tailoring care to the individual and that due to the link between complex multimorbidity and polypharmacy, patients who are prescribed ≥ 15 repeat medicines should be specifically targeted and offered an individualised structured medication review ^5^. SPPiRE is a cluster randomised controlled trial (RCT) that is assessing the effectiveness of an intervention designed to support general practitioners (GPs) in reducing significant polypharmacy and potentially inappropriate prescribing (PIP) in older patients aged ≥ 65 years with multimorbidity in Irish primary care ^6^. Details of the trial design have been described elsewhere ^6^. The study is a pragmatic cluster randomised control trial and the intervention will be delivered at GP level. Data will be collected from both the control and intervention practices at baseline and 6 months following completion of the intervention. Control practices will be paired with an intervention practice at the time of randomization to ensure similar follow up times.

The main objectives of the study are to evaluate the effectiveness of a web-supported medication review in patients over the age of 65 years with multimorbidity polypharmacy in terms of improving medicines management, addressing patient priorities, cost effectiveness and whether it would be appropriate to deliver this project on a national basis.

The Irish healthcare system has a mixture of private and public provision and funding. There are two main categories of patients. Approximately 35% of the population, those on lowest incomes, are entitled to free primary and specialty care services but pay a €1.50 co-payment for prescription items up to a maximum of €15 per month. The remainder of the population are entitled to free hospital care with some co-payments but must pay the full cost of GP services and pay for medicines up to a maximum of €125 per family per month. Income thresholds for over-70’s are substantially higher so that the majority of patients in this category are entitled to free GP care

**Figure 1.**
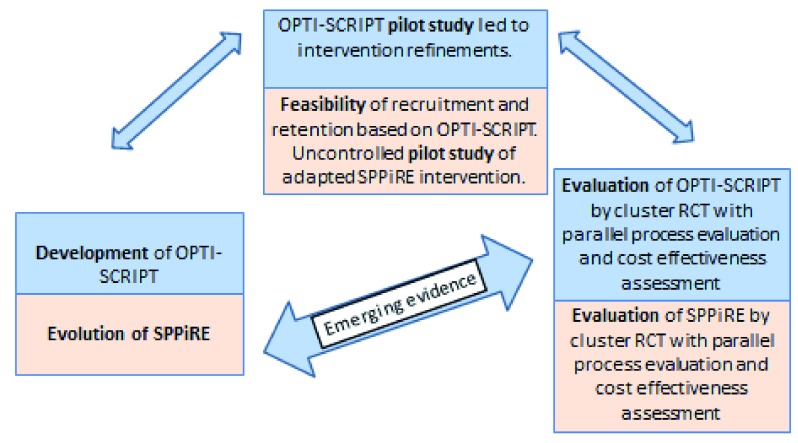
Development and evaluation of the SPPiRE intervention adapted from the MRC framework ^1^.

Briefly, the SPPiRE has been developed from previous research and current literature (see Figure 1). Our study group had previously found a complex intervention comprising academic detailing and a GP-led medicines review was effective in reducing potentially inappropriate prescribing (OPTI-SCRIPT trial) ^7^. The SPPiRE intervention evolved based on the OPTI-SCRIPT process evaluation ^8^ and emerging evidence in the area of deprescribing and high risk prescribing ^9, 10^ and was further refined following an uncontrolled pilot study of the intervention ^6^

The SPPiRE intervention will be delivered at the cluster level to intervention GPs through the SPPiRE website. The website has links to training videos and provides structure for the medication review for each individual patient, where the GP will be prompted to check for specific PIP that have been pre-selected using the updated Screening tool of older persons’ prescriptions (STOPP 2) ^11^, based on their association with preventable drug related morbidity ^12^ and their prevalence in Irish primary care ^13^, as well as recently developed and validated monitoring criteria ^14^. GPs will also be prompted to discuss and record patients’ patients’ priorities about treatment and ask them if they have concerns about their medications. Any medication changes will be at the discretion of the prescribing GP. Control practices will deliver usual care over the 6 month study period. The intervention practices will have access to training videos which will impart knowledge on polypharmacy, common potentially inappropriate prescriptions in older people, multimorbidity and treatment burden.

The components of the SPPiRE intervention are described in Table 1.

**Table 1.** SPPiRE intervention components.

SPPiRE intervention component	Description
Training videos	- demonstrate how to perform a SPPiRE medication review - describe key concepts - polypharmacy, PIP, multimorbidity and treatment burden
Medication review	Online medication review template which provides a structured process. GPs guided to: 1. Screen patient prescriptions for PIP and high risk prescribing 2. Assess the patient’s treatment priorities 3. Review each medicine with the patient 4. Agree all changes with the patient

The SPPiRE medication review has two steps

Gather and record information:

1.1 Check for potentially inappropriate prescriptions (PIPs):
  • Identify relevant drug groups
  • Record PIP if present
1.2 Address patient priorities:
  • Record patient treatment priorities
  • Consider if ongoing symptoms could be adverse drug reactions
1.3 Conduct a brown bag medication review:
  • Assess for effectiveness and side effects
  • Assess for actual drug utilisation
  • Record any concerns identified by the GP or patient

1. Agree and record changes based on information obtained in step 1:

2.1 Review identified PIP, consider suggested alternatives and record any agreed changes
2.2 Review patient treatment priorities, consider if ongoing symptoms could be adverse drug reactions and record any agreed changes
2.3 Review information input during brown bag review and record any agreed changes.

**Figure 2.**
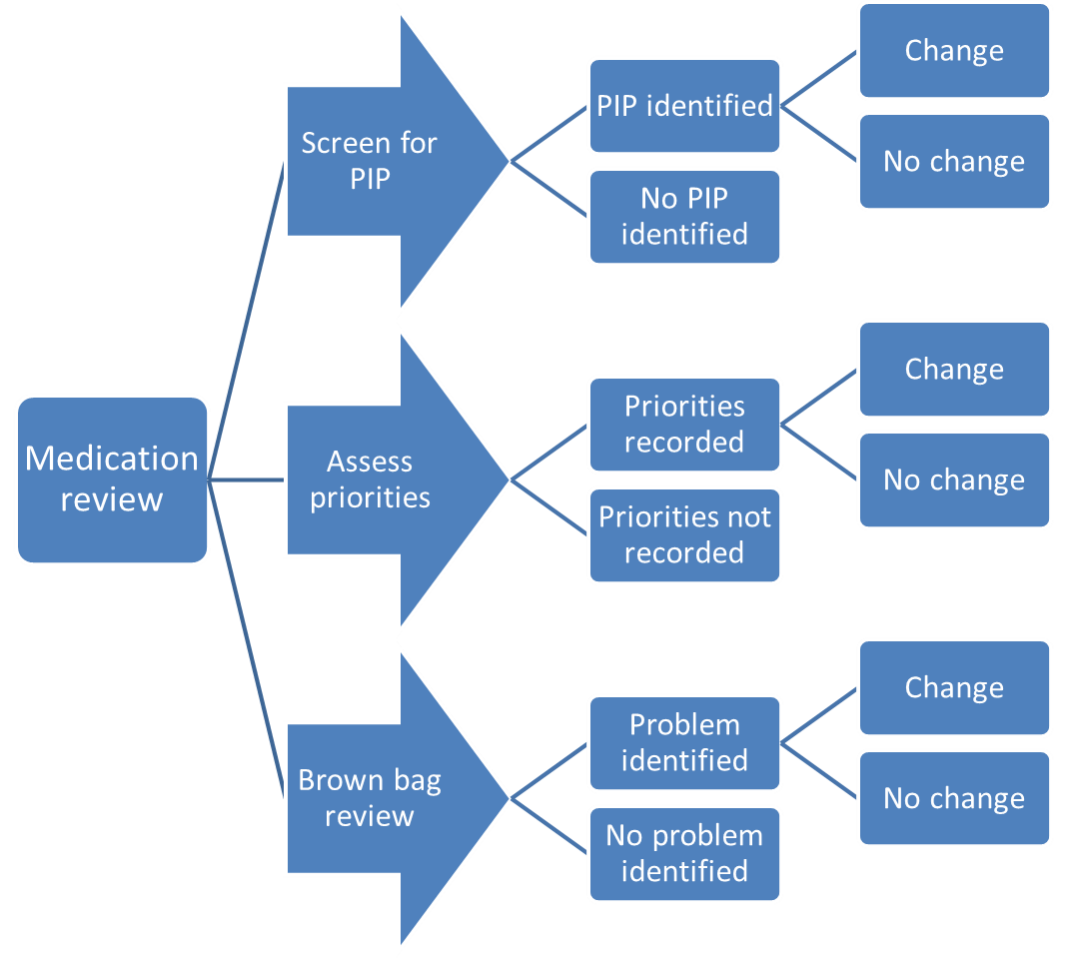
Hypothesized pathway of change for the SPPiRE medication review.

The MRC guideline on process evaluations for complex interventions advises that the various components of the intervention should be clearly described as should the mechanisms through which these components are expected to produce change ^2^. These causal assumptions may be based on theory but in the case of complex interventions are often based on common sense and past experience, as is the case with the SPPiRE intervention ^2^. Similarly recommendations from a framework for process evaluations for cluster RCTs ^3^, advises pre-specifying the hypothesized pathway of change through which the intervention is anticipated to exert its effect. Figure 2 illustrates the hypothesized pathway of change for the SPPiRE medication review. We are not testing any specific mechanisms of action or casual pathways, rather we are exploring the mechanisms through which the intervention may bring about change generically. The aim of the process evaluation will be to explore the effect of the intervention and how it was implemented.

The process evaluation will measure:

1. Use of intervention software
2. Clinical/prescribing decision made e.g. stop or start a medicine
3. Reported primary reason for decision made
4. Whether assessment of patient priorities resulted in medication change and which priorities were associated with most change
5. Immediate pre and post intervention prescription

In line with the NICE multimorbidity Guidance, GPs are also prompted to assess the patient’s treatment priorities. There is very little in the published literature on how to best to assess patient treatment priorities. A systematic review identified only one patient priority assessment tool that has been used and validated in people with multimorbidity ^15, 16^. This tool was not included as part of the SPPiRE intervention as it had not been identified at that time. The effectiveness and mechanism of effect of thepatient priotitization elements of the SPPiRE intervention will be explored in the process evaluation using both qualitative and quantitative methods.

The purpose of the brown bag medication review is to incorporate the patient’s ideas and concerns about their medicines and to identify and deprescribe medicines that may not constitute high risk PIP but are inappropriate none the less as they are ineffective or have risks that outweigh benefits in the particular individual. Qualitative work with doctors has highlighted several barriers to deprescribing, including feasibility issues, prescriber confidence and prioritization (or lack of) of deprescribing ^17^. However qualitative work with patients indicates that most would be agreeable to deprescribing medication if supported by their GP ^18^. These more nuanced areas of doctor and patient attitudes to deprescribing will be explored qualitatively in the process evaluation.

## Aims and objectives of the sppire process evaluation

**Figure 3.**
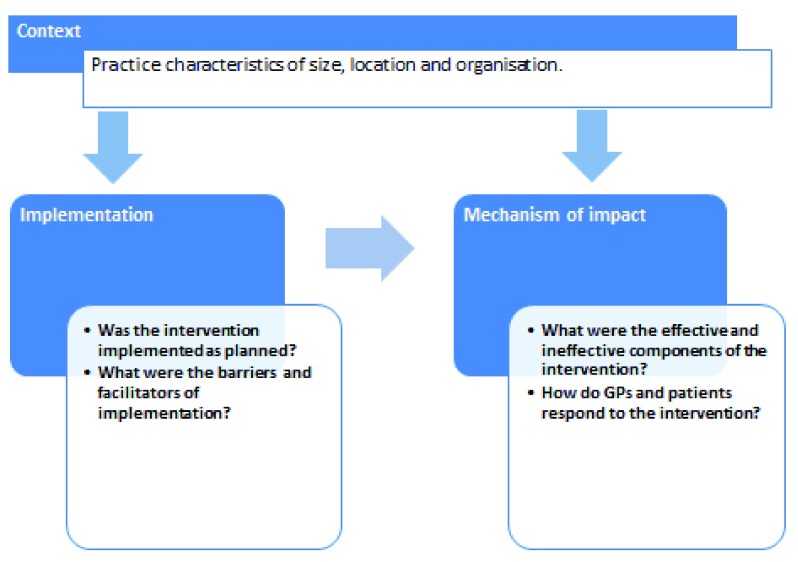
Functions of the SPPiRE process evaluation adapted from MRC guidance on process evaluations for complex interventions ^2^.

The overall aim of the SPPiRE process evaluation is to explore how and why the intervention was effective or ineffective and the potential for system wide implementation of the SPPiRE intervention in Irish primary care. Using the MRC framework, the general themes of context, implementation and mechanism of impact will be explored ^2^. Elements for reporting of process evaluations of cluster RCTs were also incorporated, as were recommendations that advise providing a detailed examination of the process of recruitment ^3^ (see Figure 3).

We will address four main themes; recruitment, implementation, mechanism of impact and the effect of the context. The specific objectives within each theme are described below.

**1. Recruitment**
  • To explore the barriers and facilitators of cluster and participant recruitment

**2. Context**
  • Does effectiveness vary between practices? If so how are practice characteristics (e.g. single handed versus group practice) associated with patient recruitment, fidelity of implementation and effectiveness of the intervention?

**3. Implementation**
  • To explore how the intervention was implemented, including the fidelity of implementation and the barriers and facilitators of implementation.

**4. Mechanism of action of the intervention**
  • Does the intervention result in change as assumed? What were the effective and ineffective components of the intervention?
  • How do GPs and patients respond to the intervention? Do they view it positively? Are there any unexpected negative or positive consequences of the intervention?

## Methods

This is a mixed-methods process evaluation which will integrate both qualitative and quantitative data to address the predetermined research objectives outlined above. Quantitative data will be analysed using Stata V13 and the qualitative data will be analysed using NVivo 12.

The SPPiRE process evaluation will run parallel to the main trial where both trial and process evaluation data collection will be contemporaneous. More detailed methods for each research theme are described below.

### Methods for research theme 1: Recruitment

***Study design.*** The overall aim of exploring recruitment is to assess the generalizability of results of the SPPiRE trial. Quantitative data pertaining to recruitment will be reported as part of the trial results according to CONSORT requirements ^19^. We will also explore why recruited practices agreed to take part and describe the barriers and facilitators of patient recruitment by individual clusters using both qualitative and quantitative methods.

***Study population.*** The study population will comprise of all recruited GP practices and patients.

***Data collection.*** Quantitative data on practice size, location and organisation will be obtained from a practice profile questionnaire ( *Extended data*) and the patient recruitment uptake will be analysed according to these factors. We hypothesize that smaller/single handed practices will have higher uptake rates.

Semi structured interviews ( *Extended data*) will be conducted via telephone with at least one GP from each recruited practice. Telephone interviewing is generally used where time or costs are issues, and evidence suggests there is little difference in the answers obtained this way ^20^.

***Plan of analysis.*** Differences between intervention practices will be described using summary statistics. The interviews will be audio-recorded (with the informed consent of the participating GPs and patients) and transcribed verbatim. Thematic analysis will be conducted by one investigator, and cross-checked by members of the research team to increase rigour and the validity of the findings ^21^.

### Methods for research theme 2: Context

***Study design.*** This theme will be explored using mixed quantitative and qualitative methods. It is hypothesised that effects will vary according to practice characteristics. The effects in smaller practices may be more concentrated compared to larger practices where some GPs may be more interested in the intervention than others. Although this may be hard to distinguish given the number of patients per practice It is hypothesised that practices that already have a rigorous system in place for managing repeat prescriptions and long-term medications may adopt the intervention more readily. It is also hypothesised that GPs working in rural areas will be more likely to intervene and change any identified problem medicines as their patients may have less ready access to hospital specialists. These hypotheses will be tested using quantitative and qualitative data.

***Study population.*** The study population will comprise of all recruited GP practices.

***Data collection.*** Practice characteristics including size (number of GP sessions per week) and location (urban, rural or mixed) will be collected on the practice profile questionnaire at baseline ^22^. Quantitative data on practice organisation (for example repeat prescribing policies and whether or not there is a practice manager) will be collected from the practice profile questionnaire.

Intervention GPs will be interviewed using semi-structured interviews and the hypotheses described above will be explored.

***Data analysis.*** Differences between intervention practices will be described using summary statistics and integrated into outcome datasets to determine if they have any effect on outcomes. Interview transcripts will be analysed using thematic analysis to further assess the validity of quantitative findings.

### Methods for research theme 3: Implementation

***Study design.*** This theme will be explored using qualitative methods. Topic guides for interviews and data analysis will be informed by the Normalisation Process Theory (NPT), a contemporary social theory that has been used to understand the factors involved in the implementation of complex interventions ^23– 25^. NPT has four major themes; coherence, cognitive participation, collective action and reflexive monitoring.

***Study population.*** The study population will comprise at least one GP from all intervention practices involved in implementing the intervention.

***Data collection.*** During the same interview at final data collection ( *Extended data*), implementation will be explored with intervention GPs. The topic guide for the interviews will include how they performed medication reviews in practice and whether they accessed the educational material, ease of use of website platform for the medication reviews and any barriers or facilitators they encountered in the process and will be structured using the NPT framework. Quantitative data will be obtained from the SPPiRE website.

***Data analysis.*** As described previously, all interviews will be recorded, transcribed verbatim and thematic analysis conducted. Thematic analysis and interpretation will be performed by one investigator and cross-checked by members of the research team to increase the validity of the findings. Website usage data will be analysed using descriptive statistics.

### Methods for research theme 4: Mechanism of impact

***Study design.*** This research theme will be explored using both quantitative and qualitative methods. Quantitative data will be obtained from the SPPiRE website. This will include data on which aspects of the medication review that the GP completed, and which aspects were most likely to result in change. Website usage data will be analyzed using descriptive statistics (means, frequencies) to summarise the types of PIP identified and the actions taken, and whether patient priorities were obtained and recorded and whether or not this resulted in change This data will be used to assess which aspect of the intervention, if any, resulted in change. Qualitative methods will include performing semi-structured interviews with both intervention GPs and patients. The intervention components and the proposed causal assumptions for change will form the basis of the topic guide for these interviews. Open and probing questions will also encourage participants to describe any unintended consequences of the intervention and their response to the intervention.

***Study population.*** The study population will comprise all intervention GPs and a purposive sample of intervention patients, to include older and younger groups, male and female patients and those on 15 medicines as well as those on over 20 medicines. A sample of 15–20 patients is proposed.

***Data collection.*** Clinical and prescribing decisions made during the medication review (e.g. stop or start medicine, refer for monitoring blood test) will be retrieved from the SPPiRE website database. Upon completion of the web guided medication review intervention, GPs will be instructed to print an immediate post intervention prescription. This will also be used to assess the most effective components of the intervention.

Qualitative data will be collected from patients and GPs using semi-structured interviews ( *Extended data*). Patient interviews will be conducted either in person or via telephone.

***Data analysis.*** Website data and post intervention prescriptions will be analysed to assess which PIP were acted upon and which aspects of the intervention were most effective. The results will be described using descriptive statistics.

The follow-up intervention prescription will be obtained at 6 months post intervention completion. We will compare it to the prescriptions at baseline and immediately after the medication review and examine:

- The number of medications prescribed
- Whether medications that were stopped at the medication review, were still discontinued at six month follow up
- Whether the patient was prescribed additional medications during the intervention or between intervention completion and follow up.

The final follow-up prescription will enable us to explore whether of any changes made during the SPPiRE website review were maintained over time.

Interview transcripts will be analysed using thematic analysis. The response to and impact of the various intervention components will be analysed.

### Final analysis

The final stage of analysis will be to draw together the findings from the quantitative analysis and qualitative analysis across the four research themes to create an understanding of why the intervention did (or did not) work in all or some contexts and identify implications for longer term implementation if appropriate.

### Dissemination of results

The results of the Trial will be presented at national and international conferences including The Association of University Department of General Practice in Ireland (AUDGPI) and Society for Academic Primary Care (SAPC) and we plan to publish the results in peer reviewed journals.

## Discussion

At the time of finalising this protocol, patient and practice recruitment to the SPPiRE trial has commenced with ongoing patient recruitment and intervention implementation.

Running the process evaluation parallel to the main trial has the advantage of reducing recall bias of participants and interview bias where researchers, aware of the results of trial outcome data, may influence participants with their preconceived ideas about the reasons for the outcomes. The main disadvantage of this approach is that it is not possible to focus on practices showing extreme positive or negative effects. We have addressed this by choosing to interview at least one GP from all recruited practices.

It is in this context that the SPPiRE intervention is being evaluated, the effect of this on both recruitment and implementation of the intervention will be explored.

If the SPPiRE intervention is effective in reducing PIP and polypharmacy in older patients, this process evaluation will help delineate what components of the intervention were most effective and provide some insight into the generalisability of the findings to Irish general practice. Similarly, if the intervention is ineffective the process evaluation will shed light on whether the hypothesized pathway of change was flawed or whether the overall context interfered with implementation of the intervention.

### Ethics approval and consent to participate

Full ethical approval for the study was granted by the Irish College of General Practitioners Research Ethics Committee (ICGP REC, SPPiRE Study). Written informed consent will be sought from all patients and GP’s participating in the study.

## Data availability

### Underlying data

No data is associated with this article.

### Extended data

Open Science Framework: SPPiRE Study, https://doi.org/10.17605/OSF.IO/HBTY5 ^26^.

This project contains the following extended data:

- Practice profile questionnaire
- Interview topic guide GPs
- Interview topic guide patients
- Follow-up data collection form
- Adverse drug withdrawal report form

Data are available under the terms of the Creative Commons Zero "No rights reserved" data waiver (CC0 1.0 Public domain dedication).
