# Supplementary material for: Study protocol for a process evaluation of a cluster randomised controlled trial to reduce potentially inappropriate prescribing and polypharmacy in patients with multimorbidity in Irish primary care (SPPiRE)
Source: HRB Open Res. 2020 Jan 21;2:20. Originally published 2019 Aug 23. [Version 2] doi: 10.12688/hrbopenres.12920.2 (PMC7016882; doi:10.12688/hrbopenres.12920.2)
Supplement: Peer review history

# Peer review: Reviewer response for version 2

**DOI:** 10.21956/hrbopenres.14078.r27093

**Published:** 2020-02-10

**Authors:** Cindy Mann

**Affiliations:** 1 Centre for Academic Primary Care, National Institute for Health Research School for Primary Care Research, Population Health Sciences, Bristol Medical School, University of Bristol, Bristol, UK

The changes in this revised article have made the design of the process evaluation much clearer. I have no further comments to make other than to wish the authors success in carrying out this interesting study.

I confirm that I have read this submission and believe that I have an appropriate level of expertise to confirm that it is of an acceptable scientific standard.

# Peer review: Reviewer response for version 2

**DOI:** 10.21956/hrbopenres.14078.r27092

**Published:** 2020-01-30

**Authors:** Aileen Grant

**Affiliations:** 1 School of Nursing and Midwifery, Robert Gordon University, Aberdeen, UK

These changes are fine with me.

I confirm that I have read this submission and believe that I have an appropriate level of expertise to confirm that it is of an acceptable scientific standard.

# Peer review: Reviewer response for version 1

**DOI:** 10.21956/hrbopenres.13998.r26777

**Published:** 2019-09-26

**Authors:** Cindy Mann

**Affiliations:** 1 Centre for Academic Primary Care, National Institute for Health Research School for Primary Care Research, Population Health Sciences, Bristol Medical School, University of Bristol, Bristol, UK

This seems overall a well-planned process evaluation of the SSPiRE cluster randomised trial, organised into four main themes of interest to the process evaluation and reporting the methods, data collection and planned analysis of each. It was mainly clearly described except that there were places where I found it a little difficult to understand because of a lack of detail. In particular it would be easier to understand the quantitative aspects of the process evaluation if the intervention were described more fully. I have looked up the published protocol referenced in the paper, which was helpful, but suggest that even so a little more detail in this paper would enhance the clarity.

 On page 4 you refer to a patient priority assessment tool and then say; ‘The effectiveness and mechanism effect of this novel aspect of the SPPiRE intervention will be explored in the process evaluation’. Does this mean that you will be using the referenced tool? If so, it would help if this was more explicit. Again, more detail about the content of the intervention would help here.

 Are there any hypothesised mechanisms of action that you are specifically evaluating? Figure 2 is more of a flow chart than an illustration of causal assumptions for change and it would be very interesting to have an additional diagram that illustrated hypothesised mechanisms of action. This would potentially give a stronger rationale to underpin the design of the process evaluation.

 On page 6 you state that ‘quantitative data will be obtained from the SSPiRE website’ and then that ‘website usage data will be analysed using descriptive statistics’. Could you explain this a bit more? Will GPs performing medication reviews be required to enter data in a template for the reviews that reflect each step in the structured process? Is the intention that researchers will then be able to tell which steps have been completed and thus evaluate the effectiveness of each component?

 You state that 55 practices will be involved but only 400 patients, presumably meaning there will be only 7-8 patients per practice. You hypothesize that ‘smaller/single handed practices will have higher uptake rates’. Does this mean uptake by the GPs or by the invited patients or both? If there are only a small number of patients taking part in the intervention per practice, it will be challenging to detect differences between practices in scale of implementation and effectiveness, but I may have misunderstood what you mean by ‘effects in smaller practices may be more concentrated compared to larger practices where some GPs may be more interested in others’ on page 6. It would be helpful if you could clarify what is meant here.

 Also on page 6, could you clarify how the post-intervention prescription will be ‘used to assess the most effective components of the intervention’?

 Finally, I noticed a couple of typos on page 6: ‘collection action’ needs changing to ‘collective action’ and ‘reflex’ needs changing to ‘reflexive’

I confirm that I have read this submission and believe that I have an appropriate level of expertise to confirm that it is of an acceptable scientific standard, however I have significant reservations, as outlined above.

# Response

**Published:** 2020-01-17

**Authors:** Karen Kyne

**Affiliations:** Beaumont Hospital and RCSI Medical School, Ireland

**Response to Reviewer 1 comments - Dr Cindy Mann**

- This seems overall a well-planned process evaluation of the SPPiRE cluster randomised trial, organised into four main themes of interest to the process evaluation and reporting the methods, data collection and planned analysis of each. It was mainly clearly described except that there were places where I found it a little difficult to understand because of a lack of detail. In particular it would be easier to understand the quantitative aspects of the process evaluation if the intervention were described more fully. I have looked up the published protocol referenced in the paper, which was helpful, but suggest that even so a little more detail in this paper would enhance the clarity.

***Author’s response:***

We have provided more detail. The text added is highlighted in red.

- On page 4 you refer to a patient priority assessment tool and then say; ‘The effectiveness and mechanism effect of this novel aspect of the SPPiRE intervention will be explored in the process evaluation’. Does this mean that you will be using the referenced tool? If so, it would help if this was more explicit.

***Author response***

We have clarified this by the addition of the following text  on page 6:

‘’A systematic review identified only one patient priority assessment tool that has been used and validated in people with multimorbidity. 15, 16 This tool was not included as part of the SPPiRE intervention. The effectiveness and mechanism effect of this novel aspect of the SPPiRE intervention will be explored in the process evaluation using both qualitative and quantitative methods.’’

- Are there any hypothesised mechanisms of action that you are specifically evaluating?

***Author response:***

We have clarified this on page 6 with the addition of text in red that no specific mechanisms of action have been prespecified for evaluation.

- Figure 2 is more of a flow chart than an illustration of causal assumptions for change and it would be very interesting to have an additional diagram that illustrated hypothesised mechanisms of action. This would potentially give a stronger rationale to underpin the design of the process evaluation.

***Author response:***

We haven’t prespecified mechanisms of action. We are examining the process and trying to establish the mechanisms of action as described in the red text added on page 6.

We do not think it would be helpful to add another diagram.

- On page 6 you state that ‘quantitative data will be obtained from the SSPiRE website’ and then that ‘website usage data will be analysed using descriptive statistics’. Could you explain this a bit more?

***Author response***

We have clarified this by the addition of the following text in red on page 9.

‘’Quantitative data will be obtained from the SPPiRE website. This will include data on GP activity (how often the website was used), the types of PIP and actions taken, and data on patient priorities.

Website usage data will be analysed using descriptive statistics (means, frequencies etc.) to summarise the use of the website and the types of PIP and actions taken.’’

- Will GPs performing medication reviews be required to enter data in a template for the reviews that reflect each step in the structured process? Is the intention that researchers will then be able to tell which steps have been completed and thus evaluate the effectiveness of each component?

**Author response**

Yes, GP’s will be required to enter the data on the template provided which will enable researchers to tell which steps have been completed and thus evaluate the effectiveness of each component.

The following text has been added on page 10 which is highlighted in red.

‘This data will be entered by the GP’s during the medication review process.’

- You state that 55 practices will be involved but only 400 patients, presumably meaning there will be only 7-8 patients per practice. You hypothesize that ‘smaller/single handed practices will have higher uptake rates’. Does this mean uptake by the GPs or by the invited patients or both?

***Author's response***

This refers to patient uptake. Our experience is that smaller practices are more likely to recruit patients, likely due to better continuity of care between individual GPs and patients.

- If there are only a small number of patients taking part in the intervention per practice, it will be challenging to detect differences between practices in scale of implementation and effectiveness, but I may have misunderstood what you mean by ‘effects in smaller practices may be more concentrated compared to larger practices where some GPs may be more interested in others’ on page 6. It would be helpful if you could clarify what is meant here.

***Author’s response***

The rationale for this is that single handed GP’s are often located in rural more remote locations, tend to be further away from hospitals and have less access to specialist services. Hence would be more likely to participate in these projects.

We agree with the statement above.

We have added the following text in red to the article ‘Although this may be hard to distinguish given the number of patients per practice.’ On page 8.

- Also on page 6, could you clarify how the post-intervention prescription will be ‘used to assess the most effective components of the intervention’?

***Author’s response***

We have clarified this with the addition of text in red on page 9 and 10.

- Finally, I noticed a couple of typos on page 6: ‘collection action’ needs changing to ‘collective action’ and ‘reflex’ needs changing to ‘reflexive’

***Author's response***

We have changed it to collective action and reflexive on page 9.

# Peer review: Reviewer response for version 1

**DOI:** 10.21956/hrbopenres.13998.r26775

**Published:** 2019-09-18

**Authors:** Aileen Grant

**Affiliations:** 1 School of Nursing and Midwifery, Robert Gordon University, Aberdeen, UK

This a well designed process evaluation however I feel there is some crucial information missing which would aid the reader in interpretation.

 This protocol could benefit from a brief description of the trial design. Is this a two arm trial? It appears there is only data collection in the intervention arm? This could result in the hawthorne effect and the process evaluation could be responsible for some of the effect observed within in the trial.  If my assumption is correct then as well as a brief description of the trial this should be acknowledged in the limitations.  

 An understanding of the primary outcome would be useful too.

 The introduction would also benefit from a brief description of the organisation of the Irish health care system.  There is an assumption the reader knows but as a Scot I do not.  This is important to interpret context. 

 Furthermore the mechanisms of action definition seems to be an over simplification.  There is not reflection of the relationship of the intervention components and the fact the whole can be greater than the sum of the effects.

I confirm that I have read this submission and believe that I have an appropriate level of expertise to confirm that it is of an acceptable scientific standard, however I have significant reservations, as outlined above.

# Response

**Published:** 2020-01-17

**Authors:** Karen Kyne

**Affiliations:** Beaumont Hospital and RCSI Medical School, Ireland

**Response to Reviewer 2 comments: Dr Aileen Grant**

- This a well designed process evaluation however I feel there is some crucial information missing which would aid the reader in interpretation.This protocol could benefit from a brief description of the trial design. Is this a two arm trial? It appears there is only data collection in the intervention arm?This could result in the hawthorne effect and the process evaluation could be responsible for some of the effect observed within in the trial.  If my assumption is correct then as well as a brief description of the trial this should be acknowledged in the limitations.  

**Author’s response:**

 More information on the trial, has been added to the text in red font on page 3.

 As per previous comments above, we have given more information on the intervention.

- An understanding of the primary outcome would be useful too.

**Author’s response:**

 More information on this has been added to the article in red font on page 3.

- The introduction would also benefit from a brief description of the organisation of the Irish health care system.  There is an assumption the reader knows but as a Scot I do not.  This is important to interpret context. 

**Author’s response:**

 A brief explanation of the Irish Healthcare system has been added to the text in red font on page 3.

- Furthermore, the mechanisms of action definition seems to be an over simplification.  There is not reflection of the relationship of the intervention components and the fact the whole can be greater than the sum of the effects.

**Author’s response:**

 We have updated this based on reviewer 1 comments as outlined by the text in red in the article.
